# Epitomic profiling and functional characteristics of pemphigus vulgaris autoantibody binding to keratinocyte M3 muscarinic acetylcholine receptor

**DOI:** 10.1016/j.jbc.2025.108434

**Published:** 2025-03-20

**Authors:** Jorge Mauricio Reyes-Ruiz, Alex Chernyavsky, Sergei A. Grando, Charles Glabe

**Affiliations:** 1 Department of Molecular Biology and Biochemistry, University of California Irvine, Irvine, California, USA; 2 Department of Dermatology, University of California Irvine, Irvine, California, USA; 3 Department of Biological Chemistry, University of California Irvine, Irvine, California, USA; 4 Institute for Immunology, University of California Irvine, Irvine, California, USA

**Keywords:** pemphigus vulgaris, autoantibody, epitomic profiling, M3 muscarinic acetylcholine receptor, acetylcholine ligand-binding pocket, keratinocytes

## Abstract

Patients with pemphigus vulgaris (PV) develop IgG autoantibodies (AuAbs) binding to keratinocyte desmogleins (Dsg), acetylcholine (ACh) receptors, mitochondrial proteins, and some other self-antigens. In this study, we identified linear and discontinuous peptide tetrameric epitope segments (ES) of M3 muscarinic ACh receptor (M3AR) targeted by different anti-M3AR AuAbs. As positive controls, we identified Dsg1 and Dsg3 ES targeted by PV sera. Healthy individuals also possessed natural antibodies targeting M3AR, Dsg1 and Dsg3 epitopes that were different from those targeted by AuAbs produced by patients with PV. The two targeted M3AR pentameric ES encompass the 10 amino acids-long epitope LSEPTITFGT included the tetramer TFGT containing Thr235 which is a part of the ACh-binding pocket. Previously, it has been demonstrated that the anti-M3AR AuAb produces an agonist-like effect on downstream signaling, but its long-term effect is receptor desensitization. In this study, we compared the functional consequences of binding anti-M3AR AuAbs that target the ACh-binding pocket with that of AuAbs that target M3AR outside of its ACh-binding pocket. While the former AuAbs induced a very high elevation of phospholipase C, inositol triphosphate and diacylglycerol, which represents an agonist-like effect, the latter AuAbs produced a much weaker signaling response. These results indicate that patients with PV develop two types of anti-M3AR AuAbs. One type attaches to orthosteric, i.e., ACh-binding, site and elicits a strong signaling response comparable to that induced by a full pharmacologic agonist, whereas another type binds to an allosteric site and elicits submaximal signaling response comparable to that induced by a partial (allosteric) agonist.

*Pemphigus vulgaris* (PV) is a potentially lethal mucocutaneous blistering disease characterized by IgG autoantibodies (AuAbs) binding to keratinocytes (KCs) and inducing devastating blisters affecting oral and/or esophageal surfaces and, sometime, also the skin. Patients with PV develop cell-cell detachment (i.e., acantholysis), blisters and non-healing erosions due to suprabasal split within the epidermis. The majority of patients with PV have AuAbs against desmoglein (Dsg) 3 ± 1, which are believed to cause acantholysis (reviewed in (1, 2)). However, on average 10% of patients with acute PV having anti-keratinocyte AuAbs detectable by direct and/or indirect immunofluorescence are negative for Dsg1/3 ELISA (reviewed in (3)). Anti-keratinocyte AuAbs in patients with anti-Dsg1/3 AuAb-negative PV are pathogenic, because their IgGs can induce skin blistering in neonatal mice due to suprabasal acantholysis (4).

Among known species of non-Dsg1/3 anti-keratinocyte AuAbs, the AuAbs against M3 subtype of the muscarinic class of acetylcholine (ACh) receptors (M3AR) have an important role, as suggested by the following lines of evidence: (i) proteomic studies demonstrated M3AR AuAbs in a large number of PV sera (5, 6); (ii) the anti-M3AR AuAb titer correlates with disease stage of PV and titer of anti-Dsg3 AuAb (4, 7); (iii) absorption of PV IgGs on recombinant M3AR prevents skin blistering in the passive transfer of AuAbs model of PV in neonatal mice and the acantholytic activity of pre-absorbed PV IgGs can be restored by adding the eluted AuAb (4); and (iv) acantholysis and Nikolsky sign in mouse skin (i.e., erosions induced by gentle rubbing with a pencil eraser) can be induced by anti-M3AR AuAb in combination with two other AuAbs affinity purified from non-Dsg PV sera (4, 8). M3AR is predominantly expressed in the lowermost epidermal layer wherein it regulates vital function of KCs (reviewed in (9, 10)). The epitopes of M3AR targeted by pathogenic anti-M3AR AuAbs in PV, however, remain unknown.

The M3AR is preferentially coupled to activation of pertussis toxin–insensitive G proteins of the Gαq/11 family. The binding of ACh to M3AR induces a conformation change in the receptor that promotes association with and activation of the Gq protein by exchanging GTP for GDP on the Gα subunit. The subsequently released Gα subunit activates phospholipase C (PLC) that hydrolyzes phosphatidylinositol 4,5-bisphosphat into inositol triphosphate (IP3) and diacylglycerol (DG). IP3 binds to IP3 receptors on the endoplasmic reticulum, releasing Ca^2+^ from intracellular stores, whereas DG activates protein kinase C. We have recently demonstrated that chronic exposure of KCs to anti-M3AR PV AuAbs interrupts the physiologic regulation by endogenous ACh thereby contributing to acantholysis (11). Binding of anti-M3AR AuAbs to KCs exhibit an agonist-like effect that leads to receptor desensitization (a.k.a. tachyphylaxis), which, in turn, abolishes the physiologic regulation of KCs by auto/paracrine ACh, so that the net pathobiologic effect of anti-M3AR AuAbs is an antagonist-like (11). The agonistic AuAbs have been demonstrated in a variety of human diseases (reviewed in (12)).

Using M3AR^−/−^ mice as a surrogate model of M3AR desensitization, we have demonstrated that the skin of mice lacking M3AR and the skin of patients with PV both display an altered epidermal morphology, such as the shrinkage of basal KCs, the proliferation of nucleated basal cells, and the augmentation of intercellular spaces (8, 13, 14, 15). These morphologic changes are associated with upregulation of cell proliferation genes and downregulation of genes contributing to epidermal differentiation, extracellular matrix formation, intercellular adhesion, and cell arrangement. Indeed, differential gene expression analysis revealed that M3AR inactivation is associated with prominent downregulation of genes contributing to extracellular matrix formation, intercellular adhesion, and cell arrangement (15).

The goal of this study was to identify specific M3AR epitopes targeted by pathogenic anti-M3AR AuAbs developed by patients with acute PV. Toward this end, we employed the epitomic profiling approach that allows us to obtain detailed information about the sequence specificity of AuAbs recognizing both linear and discontinuous epitopes by identifying two or more sequence segments that bind to the same antibody (16, 17). The results demonstrate that patients with PV develop two types of anti-M3AR AuAbs, one targeting the ACh-binding pocket and another one targeting M3AR outside of its ACh-binding pocket. While the former AuAbs induce a very high elevation of second messengers, which is consistent with an agonist-like effect, the latter produce a much weaker response. The results also revealed that healthy donor controls (HD) develop natural antibodies (Nab) to M3AR that may play a protective role. These findings, therefore, shed a new light on the immunopharmacology of pemphigus autoimmunity against its major targets on the keratinocyte cell membrane and suggested novel therapeutic approaches.

## Results

### Differential epitope targeting of self-antigens by IgGs from patients with PV vs. HD

**Figure 1:**
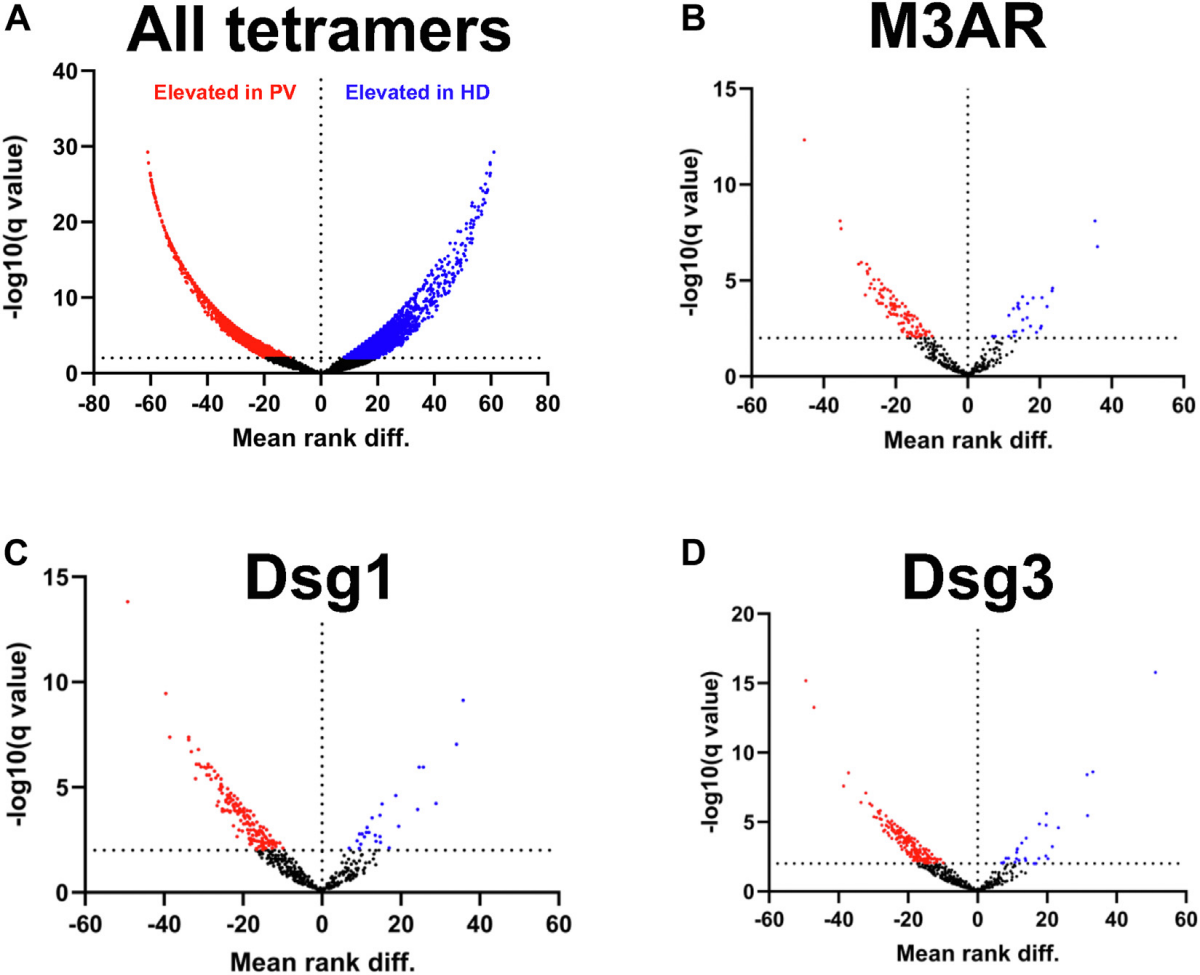
**Volcano plots of antibody specific tetramers significantly elevated in PV and HD cohorts.** Individual data points represent one of the differentially expressed tetramers in the multiple comparison analysis (q < 0.02). ES elevated in PV are shown in *red* on the *left* side of the plot and those elevated in HD are shown in *blue* on the *right* side. We used the nonparametric multiple comparisons Mann-Whitey *U* test with a threshold of 0.02 after comparison FDR correction. The q value is the *p* value after correction for FDR. (*A*) Total ES elevated in the PV and HD cohorts. Tetramers of M3AR (*B*), Dsg1 (*C*) and Dsg3 (*D*) targeted by AuAbs in the PV and HD cohorts.

To identify the differentially expressed epitope segments (ES) associated with the acute phase of PV, we immunoselected random seven mers from a phage display library using three sequential pannings followed by elution and amplification of the immunoselected phage. An average of approximately seven million sequence reads was obtained for each serum sample. The seven mer sequences were tiled in four sequential overlapping tetramers and the frequency of each tetramers was counted for each sample. The purpose of initial tiling in tetramers is to distinguish antibody-specific sequences from non-binding random sequences. We determined that tetrameric ES were the optimal compromise between sensitivity and specificity and that tiling in longer segments are not necessary because longer linear segments are readily apparent as sequentially overlapping tetramers (i.e., two overlapping tetramers is a pentamer while three overlapping tetramers is a hexamer). We found 20,584 ES elevated in the PV cohort and 6058 ES elevated in the HD cohort (*p* < 0.02) (Fig. 1*A*).

When measuring the specific binding of PV IgGs to M3AR, which contains 586 tetramers, we found that 145 tetramers were targeted by AuAbs (Fig. 1*B*). As positive controls to validate our epitomic approach, we also measured binding of PV AuAbs to the ES for Dsg1 and Dsg3 epitopes, which are well-known targets of PV autoimmunity (Fig. 1, *C* and *D*). Dsg1 protein contains 1046 tetramers, of which 278 were targeted by PV AuAbs, whereas in Dsg3 protein contains 996 tetramers, of which 240 were targeted. In all three proteins, the targeted ES covered approximately two-thirds of the sequence, indicating that a large number of IgG AuAbs target these proteins in patients with patients with PV. Since the M3AR protein shares only two tetrameric ES with Dsg1 and four with Dsg3, targeting M3AR by cross-reacting anti-Dsg1/3 PV AuAbs appears to be very unlikely.

Unexpectedly, we also found that HD possess elevated levels of NAbs targeting the M3AR, Dsg1, and Dsg3 epitopes which, however, were different from those targeted by AuAbs produced by patients with acute PV (Fig. 1). We identified 24 ES for M3AR targeted by NAb produced by HD, and 28 ES and 29 ES for Dsg1 and Dsg3, respectively, suggesting that some NAbs may be protective from PV.

These results indicated that epitomic profiling is an adequate approach to evaluate the structural elements of the most common self-antigens targeted by pemphigus autoimmunity and that Dsg1, Dsg3 and M3AR are all targets of large numbers of different antibodies associated with PV.

### The M3AR ES differentially associated with acute PV

**Figure 2:**
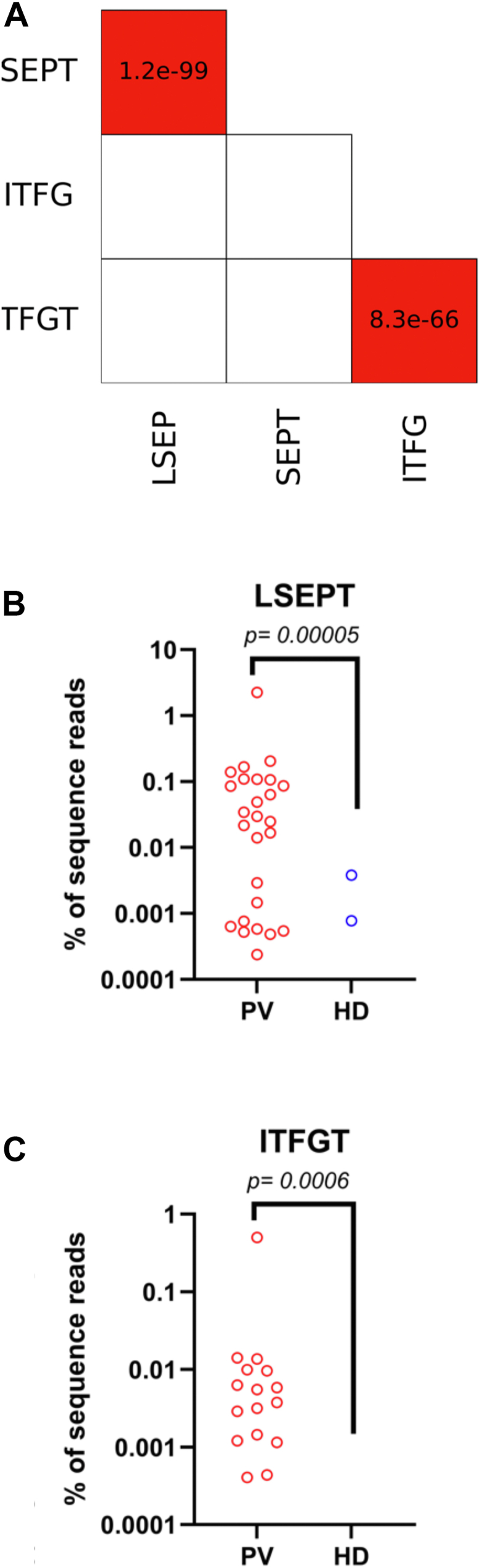
**Epitope segments differentially associated with acute PV.***A*, Pearson correlation matrix of statistically significant ES belonging to the pentamers LSEPT and ITFGT. *p* values below 0.05 are *shaded red* and displayed in the cell. *B* and *C*, scatter plots showing individual data points of the times the pentamers LSEPT (*B*) and ITFGT (*C*) were found in the PV compared to those found in the HD cohorts. Note, the values for the ES of ITFGT in the HD cohort are zero.

**Figure 3:**
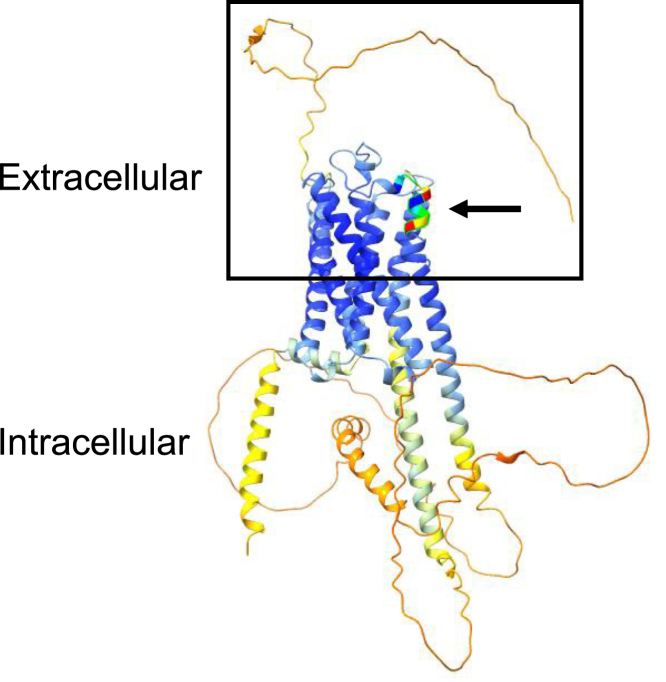
**The AlphaFold 3D model of M3AR.** Colored region marked by the *arrow* is the LSEPTITFGT epitope (amino acids 226–235) located on the border of the second extracellular loop and the fifth transmembrane helix. Note, the anti-M3AR PV AuAbs target the tetramer TFGT containing Thr235 which is a part of the ACh-binding pocket (*arrow*).

Next, we sought to identify specific M3AR epitope(s) targeted by anti-M3AR AuAbs from patients with acute PV. The four most common tetramers targeted anti-M3AR PV AuAbs were found to be LSEP, SEPT, ITFG and TFGT (Fig. 2*A*) forming two adjacent pentameric ES LSEPT (40% sera) and ITFGT (21% sera). Inspection of the heptameric sequences indicated that the two overlapping tetramers are in fact a pentamer. To map the targeted epitopes, we used structures reported on AlphaFold (https://alphafold.ebi.ac.uk/entry/P20309). Remarkably, the targeted pentamers encompass the 10 amino acids-long sequence segment LSEPTITFGT (amino acids 226–235) located on the border of the second extracellular loop (amino acids 208–229) and the fifth transmembrane helix (amino acids 230–252) (Fig. 3). This 10 residue linear segment is recognized by two different AuAbs; one that recognizes the pentamer formed by the two overlapping tetramers LSEP and SEPT and a distinct pentamer formed from ITFG and TFGT. Since there are no significant ES overlapping the junction between LSEPT and ITFGT, and there are no significant Pearson correlations between the tetramers comprising LSEPT and ITFGT, they are likely distinct epitopes recognized by different AuAbs (Fig. 2*A*). Notably, anti-M3AR PV AuAbs targeted the pentamer ITFGT containing Thr235 which is a part of the ACh-binding pocket. The ACh-binding pocket of M3AR is comprised by the following amino acids: Asp148, Ser152, Asn153, Phe222, Ile223, Thr235, Ala236, Ala239, Trp504, Asn508, Val511, Leu512, Cys533, and Tyr534 (18). Other statistically significant M3AR pentamers targeted by AuAbs in acute PV were TLHNN (33% sera) and VHPTG (29% sera).The fact that each particular M3AR pentamer was not targeted by 100% of PV sera was not surprising, because we observed the same paradigm with the Dsg1 and Dsg3 sequences. For example, the most commonly targeted pentamer of Dsg1 (RALNS) was recognized by 52% of sera and that of Dsg3 (RALNA) by 43% of PV sera.

These results indicated that direct binding of PV AuAbs to the ligand-binding pocket of keratinocyte M3AR can mimic the biologic effect of the natural agonist ACh.

### Effects of PV IgGs on M3AR-mediated downstream signaling in human KCs

**Figure 4:**
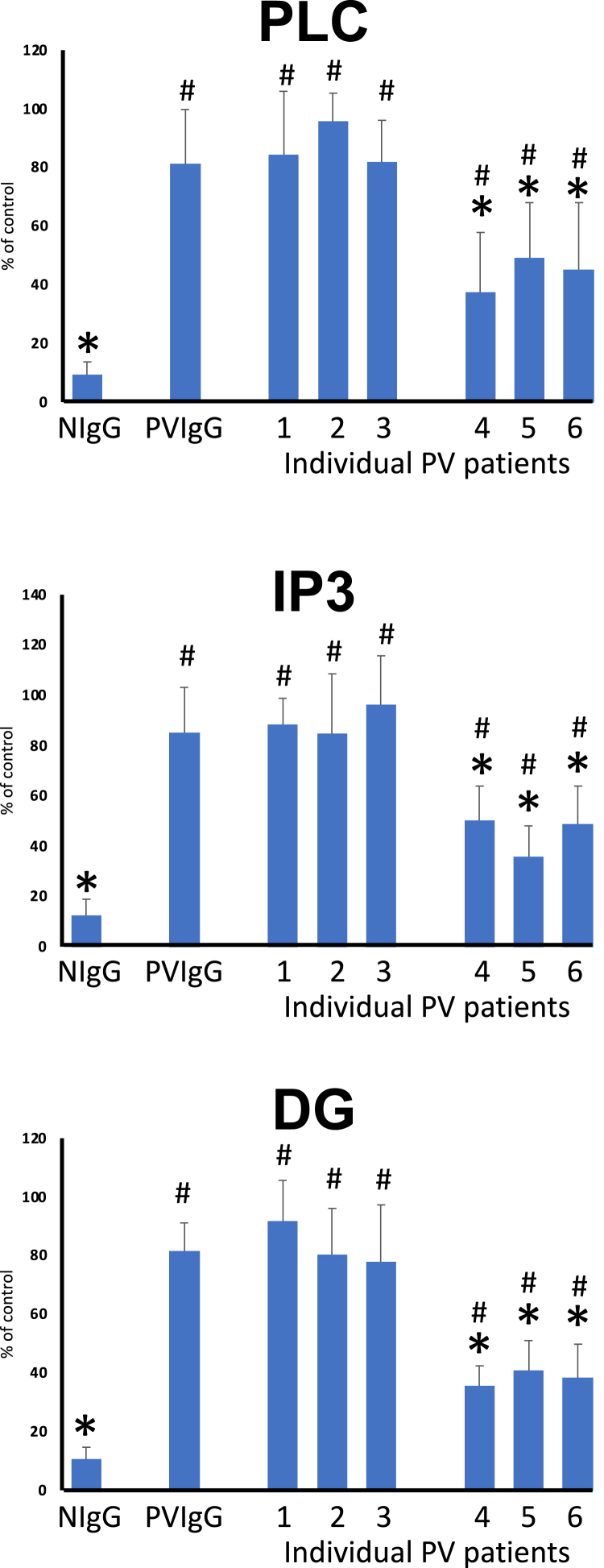
**Analysis of the effects of anti-M3AR AuAbs on receptor-mediated downstream signaling in human KCs.** The monolayers of Het-1A cells were preincubated for 1 h in the growth medium containing 1 μM muscarine to activate M3AR (taken as 100%) or 1 mg/ml of PV or HD serum IgG fractions. After incubation, the monolayers were washed, lysed, and used in the PLC, IP3 and DG assays performed in accordance with the manufacturer's instructions. Noteworthily, there were no appreciable differences in clinical course of PV between the groups of patients that did versus did not have AuAbs against the ITFGT epitope. All patients had the mucocutaneous variant of PV and were successfully treated with the multidrug protocol described by us elsewhere (55). Asterisk = *p* < 0.05 compared to control; pound sign = *p* < 0.05 compared to intact cells representing the baseline activity of M3AR. NIgG = normal human IgG.

Next, we sought to determine the functional consequences of binding of anti-M3AR PV AuAbs that target the ACh-binding pocket vs. those binding outside of the ligand-binding site. Toward this goal, we compared the effects of IgGs isolated from PV sera that had highest counts for ITFGT, which is proportional to serum concentration of an AuAb (17), with those that had zero such counts. Since our previous studies demonstrated that an immediate immunopharmacologic effect of anti-M3AR PV AuAbs is agonist-like (11), we measured changes in the concentration of the second messengers PLC, IP3 and DG in monolayers of human KCs. As a positive control, we used the M3AR agonist muscarine. Exposure to IgGs from patients with PV having AuAbs against the ITFGT epitope (i.e., patients ##1, 2 and 3 on Fig. 4) caused significant elevation of PLC, IP3 and DG, i.e., produced a strong agonist-like effect that was similar to that of muscarine. In marked contrast, AuAbs that targeted M3AR outside of its ACh-binding pocket (i.e., patients ##4, 5 and 6 on Fig. 4) produced a much weaker response.

These results indicated that patients with acute PV develop two types of anti-M3AR AuAbs that possess their own intrinsic activity to activate the receptor in the absence of the natural agonist ACh. One type attaches to orthosteric, i.e., ACh-binding, site and elicits a very strong signaling response comparable to that produced by a full pharmacologic agonist, whereas another type binds to an allosteric site and elicits submaximal signaling response comparable to that produced by a partial (allosteric) pharmacologic agonist.

## Discussion

Human epidermal KCs of both oral mucosa and skin express all five subtypes of muscarinic ACh receptors (mAChRs), M1-M5, that mediate the physiologic control of vital keratinocyte functions such as proliferation, migration, and cell-cell and cell-substrate attachments by an auto/paracrine ACh (reviewed in (9, 10)). M3AR plays the pivotal role in mediating ACh control of basal cells promoting their adhesion and differentiation and suppressing proliferation (15). Remarkably, the cholinergic drugs that mimic the pharmacologic effect of ACh abolish the PV IgG-induced acantholysis in a keratinocyte monolayer (19) and skin of neonatal mice (20) and, most importantly, alleviate PV symptoms in patients resistant to prednisone (20, 21, 22, 23). Hence, the elucidation of the immunopharmacology of pemphigus autoimmunity against keratinocyte M3AR may lead to novel and safer treatments of patients with PV.

Although the presence of AuAbs to mAChRs in PV has been known for >30 years (24), specific targeting of M3AR was discovered relatively recently in proteomic studies independently performed by our (5) and Dr Sinha's groups (6). Recognition of the whole M3AR protein has been demonstrated in our previous experiments, in which PV IgGs immunoaffinity purified on the synthetic sequence of M3AR visualized a single protein band with expected molecular mass in immunoblots of homogenized monolayers of human KCs (11). Like Dsg1 and Dsg3, we find that greater than 50% of the total M3AR protein is the target of PV-specific AuAb binding sites. Since it is possible to obtain millions of sequence reads from a single serum sample using the epitomic profiling, we used this approach in the present study to determine the set of tetrameric peptide ES immunoselected by the IgGs from patients with acute PV and Nab present in blood samples of age/sex-matched healthy individuals. We have previously demonstrated that the sequence frequency is a reasonable surrogate for the relative AuAb concentration in serum (17). We chose tetramers because we had previously found that four residues is commonly observed as the size of an ES (17) and because tetramers provide a higher degree of specificity or uniqueness than trimers (25). After mapping the statistically significant ES to the target protein, we build larger linear epitopes by combining highly correlated overlapping tetramers to make pentamers, hexamers, *etc.* The filtering approach employed by us in this study removes tetramers that have the same frequency in the antibody selected sequences as in the unselected library. It has the advantage of removing both tetramers derived from non-specific binding of phage and also non-specific tetrameric segments from the heptameric random sequences outside of tetramers that bind specifically to the antibody. In the pilot studies, we have tested the filtering algorithm by tiling random sequences from the unselected library and filtering them. We found that the filter removes the tetramers derived from unselected sequences. The remaining unfiltered random sequences appear to be derived mostly from sequences that are over-represented in the library, perhaps due to artifacts of library construction or preferential amplification of some sequences. The results we obtained with epitomic profiling closely match those that we have previously reported using protein array profiling (5) although the epitomic profiling we report here provides more details about the number of antibody binding sites on the protein and their location.

In keeping with our previous observation of the mimetic, or an agonist-like, effects of anti-M3AR PV AuAbs (11), which might result from IgG interaction with an active site of M3AR in imitation of the natural ligand ACh, in the present study we demonstrated direct binding of the PV AuAbs to the ACh-binding pocket. Functional abnormalities due to IgG antibody to the second extracellular loop of M3AR have been also reported in experiments with rabbits immunized with the M3R^228-237^ peptide involving the ACh-binding pocket (26). A higher elevation of the concentration of the second messengers PLC, IP3 and DG in KCs treated with the orthosteric compared to the allosteric anti-M3AR AuAbs indicated that the former AuAb indeed induced conformational changes of the receptor that are similar to those occurring due to binding of the natural agonist ACh. The facts that binding of PV IgGs to KCs can activate PLC, elevate DG and IP3 levels, upregulate PKC activity and increase the concentration of the intracellular free Ca^2+^, and that these signaling events play an important role in pemphigus pathophysiology by altering the cell-cell adhesion of KCs are well known (27, 28, 29, 30). Although various keratinocyte cell membrane proteins targeted by PV IgGs might trigger similar signaling events, the presence of an orthosteric anti-M3AR AuAb in the serum of patients with acute PV helps explain and reconcile previously reported findings.

In addition to PV, autoimmunity to M3AR has been reported in patients with other diseases, including primary Sjögren's syndrome (31), primary biliary cholangitis (32), systemic sclerosis (33), myasthenia gravis (34), thymoma (34) and chronic fatigue syndrome (35). Taking into consideration the ubiquitous presence of M3AR in human tissues and organs and the versatile functions regulated by M3AR in various cell types, it remains unknown why anti-M3AR autoimmunity in each of these diseases selectively affect function of the relevant cell type. Several explanations are possible. First, the pathogenic action of anti-M3AR AuAbs alone may be insufficient to irreversibly inactivate relevant cell function, such as adhesion in PV, and would require the synergistic actions on the part of other types of anti-keratinocyte AuAbs, i.e., those targeting Dsg1 and/or Dsg3 (7) or desmocollin 3 together with secretory pathway Ca^2+^/Mn^2+^-ATPase isoform 1 (4). Second, the humoral autoimmunity against M3AR may be insufficient to induces clinical signs of the disease and would require contribution on the part of effectors of cell-mediated cytotoxicity, like in primary Sjögren's syndrome (36, 37). Third, since anti-M3AR AuAbs bind to a discontinuous epitope comprised of sequence segments brought together in steric proximity by intramolecular disulfide bonding (38), the conformational epitopes of self-antigens in each disease may be unique to the targeted cell type.

The fact that each particular M3AR ES was not targeted by 100% of PV sera was not surprising, because we observed the same paradigm with the Dsg1 and Dsg3 sequences. Since every PV patient developed AuAbs to several tetramers of all three targeted molecules under consideration, it appears that more than just a single AuAb is required to elicit functional response, as it was predicted by our “multiple hit” hypothesis of pemphigus immunopathogenesis (39).

The development of a better treatment regimen for patients with PV is urgently needed, because of a relatively high morbidity from currently used treatment protocols (40, 41, 42, 43, 44, 45). Further studies of the immunopharmacologic action of anti-M3AR AuAb should lead to better understanding of the complex pathophysiology of this serious autoimmune disease and identification of new specific treatment options utilizing therapeutic interventions at M3AR. For instance, blocking the functional sequelae of binding of a single type of pathogenic AuAb, such as one targeting the ACh-binding pocket on M3AR, may be sufficient to restore keratinocyte adhesion, and, most importantly, treat patients with PV without the need to use high doses of systemic steroids and/or potentially dangerous immunosuppressive drugs. In this regards, it is important to emphasize that the results of the epitomic profiling revealed that healthy individuals possess NAb targeting the M3AR, as well as Dsg1 and Dsg3, epitopes that are different from those targeted by AuAbs to respective proteins produced by patients with acute PV. This rather unexpected observation helps explain, in part, a rapid therapeutic effect of intravenous IgG (i.e., IVIg) reported in some patients with PV (46). Perhaps, the most effective IVIg batches contained Nab to M3AR and/or other known pemphigus self-antigens, such as Dgs1 or Dsg3, whose binding to non-functional epitopes of the targeted molecules protected them from pathogenic AuAbs. Presumably, such Nab successfully competed with pathogenic AuAbs. Indeed, it has been demonstrated by other workers that anti-Dsg3 AuAbs from different patients display differences in the target sequence residues that are critical for Dsg3 function (47, 48). Furthermore, it is well-known that the low-titer (i.e., 1:10) IgG Nab staining the epithelial substrates in a pemphigus-like pattern are present in donor population (49). Hence, identifying conformational epitopes for the M3AR targeted by pathogenic anti-M3AR AuAbs produced in patients with PV vs. protective anti-M3AR Nab produced in recovered patients with PV and HD is an important and challenging topic of autoimmunity that will lay a groundwork for a breakthrough treatment approach of an autoantibody-mediated disease. The epitomic analysis may thus become a rapid and facile means for selecting the IgG donors with protective AuAbs.

## Experimental procedures

### Test sera and IgG fractions, Het-1A cells, and reagents

We used serum specimens from patients with acute PV and healthy donors (HD). This research had been approved by Institutional Review Board of University of California Irvine (2009–7049). The PV IgG and normal IgG (NIgG) serum fractions were isolated by FPLC protein G affinity chromatography (50). The Het-1A cell line—an established clonal population of SV40-immortalized human esophageal squamous epithelial cells (i.e., KCs) (51)—was purchased from American Type Culture Collection (catalog no. CRL-2692) and propagated in the Clonetics brand bronchial cell medium without retinoic acid (Cambrex Bio Sciences), as detailed by us elsewhere (52). The ELISA kits for measurements of PLC, IP3 and DG were purchased from MyBioSource, Inc. All ELISA assays were performed following protocols provided by the manufacturers in triplicate (*n* = 3).

### Epitomic profiling

We performed epitomic profiling on 122 human protein A purified IgG antibody samples, 74 IgG samples from patients with acute PV and 48 IgG samples from HD as described by us in detail elsewhere (17). Briefly, 10 μl of serum IgGs were used to immunoselect random sequences from a library of a diversity of 1 × 10^9^ heptapeptides expressed in the filamentous M13 phage (NEB PhD7, Catalog #E8211S). Unique sequences were identified and counted and output as a two column format for each sample that contains the unique 7 amino acid sequence and the number of times it was observed. These sequences were then tiled into overlapping tetramers using the Epitomic Profiling program and the frequency of each tetramer in each sample was counted similar to a previously published “K-tope” approach that tiled the immunoselected sequences by pentamers (53). After tiling the seven mer sequences into overlapping tetramers and counting their frequency, the non-specific tetramers, defined as tetramer sequences that have a frequency statistically indistinguishable from the frequency of the same tetramer sequence in the unselected library in the absence of antibody, were removed. The expected frequency was calculated based on the observed frequency of the individual amino acids in the library and any tetramer with an observed frequency indistinguishable from the frequency of the unselected tetramers according to Chi square analysis with a significance criterion of *p* < 0.05 was assigned a value of 0. The filtered data were output as a table of 160,000 possible tetramers and their frequencies for all samples in the PV and HD populations. In order to normalize the samples, the number found for each tetramer was divided by the total number of tetramers in the sample and the resulting number represented the percentage of tetramer in the whole sample. To identify potential discontinuous epitopes, the tetramers found to be statistically significantly elevated were used to create Pearson correlation matrices. The correlation among contiguous tetramers revealed pentamers or hexamers recognized by the same antibody.

### Statistical analysis

The data from PLC, IP3 and DG measurements were analyzed using ANOVA against an α level of 0.05 and presented as mean ± SD. In the epitomic profiling experiments, the antibody specific tetramers were compared between the PV and HD populations by nonparametric Mann-Whitney *U*-test with adaptive two-stage step-up multiple comparison false discovery rate (FDR) correction (54). This method of analysis was chosen because when we measured the distribution normality of the number of tetramers found on every individual sample using the D'Agostino & Pearson, Anderson-Darling and Kolmogorov-Smirnov tests, all three returned a non-normal result. The data are output in a file containing the ES, *p* value, mean rank in PV, mean rank in HD, mean rank difference, U statistic value and q value. The q value was set as a threshold to 0.02. The resulting q values and the rank differences between groups were used to generate volcano plots. All statistical analyses were done on GraphPad Prism 10.

## Data availability

The data sets used for the present study are available from the corresponding author upon reasonable request.

## Conflict of interest

The authors declare that they have no conflicts of interest with the contents of this article.
